# Spatial Distribution of Topmouth Gudgeonis *Pseudorasbora parva* Under Climate Change by Ensemble Models

**DOI:** 10.1002/ece3.73612

**Published:** 2026-05-05

**Authors:** Hao Li, Wenqian Sun, Wen Xiong, Tao Ju, Wenhui Wang, Li Tang, Zhengxiang Wang, Xin Gao, Lei Pan, Xianghong Dong, Yu Peng

**Affiliations:** ^1^ Hubei Key Laboratory of Regional Development and Environmental Response, Faculty of Resources and Environmental Science Hubei University Wuhan People's Republic of China; ^2^ College of Life Sciences Hubei Normal University Huangshi People's Republic of China; ^3^ Guangxi Academy of Marine Sciences Guangxi Academy of Sciences Nanning People's Republic of China; ^4^ The Key Laboratory of Aquatic Biodiversity and Conservation of Chinese Academy of Sciences, Institute of Hydrobiology Chinese Academy of Sciences Wuhan People's Republic of China; ^5^ College of Animal Science Guizhou University Guiyang People's Republic of China

**Keywords:** climate change, ensemble model, freshwater invasive species, species distribution models, topmouth gudgeon

## Abstract

Climate change may exacerbate biological invasion by expanding the distribution of invasive species. *Pseudorasbora parva* (Temminck & Schlegel, 1846) is a prevalent invasive fish species. It has posed significant threats to biodiversity and ecosystems in over 40 countries across Asia, Europe, and Africa. To avoid or mitigate its threats, it is necessary to evaluate its invasion risk. Species distribution models (SDMs), using occurrence data and bioclimatic factors, serve as critical tools for evaluating biological invasion risks. ANN, FDA, GAM, GBM, MARS, and RF are prominent individual algorithms. Ensemble models are generally considered better than individual algorithms as they can effectively reduce uncertainties. However, no study has yet used ensemble models to forecast the global distribution of *P. parva* under future climate change. Therefore, the aim of this study was to use ensemble SDMs, combined by above‐mentioned six individual algorithms, to predict the global potential suitable habitats and influencing factors of *P. parva* under current and future climate change. Global occurrence data for *P. parva* were collected from online biodiversity platforms and literature databases. Bioclimatic variables were from WorldClim and ENVIREM. The results showed as follows: (1) The ensemble model demonstrated excellent performance with an AUC of 0.993; (2) The two bioclimatic factors exerting the most significant influence on *P. parva* distribution were the maximum temperature of the coldest month and isothermality, contributing 51.362% and 18.279% respectively; (3) The distribution of *P. parva* in the current period, as generated by the ensemble model, revealed that the primary invasion areas are concentrated at latitude 22°–55° N; (4) Future projections under various climate scenarios indicated an overall range expansion, with high‐latitude or high‐altitude regions becoming increasingly favorable. These findings suggest that the future global expansion trend of *P. parva* should not be ignored and effective management policies for its invasion should be provided.

## Graphical abstract

This study predicted the distribution of *Pseudorasbora parva* , covering the ensemble model, global scale, and climate change. The overall trend shows that the potential suitable areas of *P. parva* will further expand, with high‐latitude or high‐altitude regions becoming more conducive to its survival. The urgent need for proactive monitoring and management strategies to mitigate the ecological impacts of *P. parva* under ongoing climate change were highlighted.

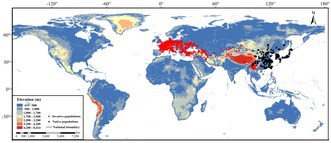

## 1 Introduction

Climate change, biological invasion, and human activities are the three critical challenges confronting global ecosystems today (Hulme 2017; Zhou and Gu 2024; Hulme 2025). Of these, the biological invasion under the background of climate change is of particular concern, which has attracted increasing scientific attention (Biancolini et al. 2024; Hulme 2025). Up to now, there have been many studies indicating that climate change might facilitate biological invasion (Osland and Feher 2020; Yan et al. 2022; Sun et al. 2025). As for the reasons, invasive species generally exhibit superior environmental adaptability and disturbance tolerance compared to native species (Yang et al. 2023). What's more, climate‐induced perturbations, such as rising temperatures, will disrupt the natural barriers of native ecosystems against invasive species and simultaneously create new suitable habitats for them (Dawson et al. 2011). For instance, invasive species previously constrained by low temperatures may expand their ranges to higher latitudes or elevations due to global warming (Dong et al. 2020; Zhao 2024). Thus, in order to inform effective management policies for biological invasion, it is necessary to comprehensively consider various factors leading to it, especially to fully understand the distribution patterns of invasive species under current and future climate change (Liu et al. 2019; Bueno et al. 2023).

Topmouth gudgeon is *Pseudorasbora parva* (Temminck & Schlegel, 1846), one of the most invasive fish species around the world, and is native to China, Japan, Korea, the Korean Peninsula, and Russia, or East Asia broadly (Lin et al. 2024). Due to aquaculture, it was accidentally introduced into Europe through mixed farming with commercial fish in the 1960s (Gozlan et al. 2010; Baltazar‐Soares et al. 2020). It has since undergone extensive spread and established stable wild populations in over 40 invaded countries across Asia, Europe, and Africa, manifesting a pan‐continental invasion nowadays (Ganjali et al. 2021). The invasion of *P. parva* significantly interfered with native species through competition (e.g., on food, habitat), egg predation, and parasite transmission, ultimately causing negative impacts on ecological, economic, and human health (Balzani et al. 2020). Experience is telling us that once an invasive population is established, it is normally prohibitively expensive and frequently unattainable to eradicate. Thus, proactive prediction is widely recognized as a more effective method (Liu et al. 2019). Accordingly, it is necessary to choose an appropriate method to predict the target species' future distribution.

Species distribution models (SDMs) are popular tools to predict the potential geographic distributions of invasive species, and there were a mass of studies worldwide using SDMs to predict the potential suitable habitats of invasive species under future climate change, aiding in biodiversity conservation and invasive species management (Forester et al. 2013; Dong et al. 2020; Zhao et al. 2021). It is worth highlighting that, compared to individual algorithms, ensemble models are generally recognized as possessing superior generalization capabilities, as they can effectively reduce uncertainties (Dong et al. 2020). However, after carefully sorting through the relevant literature, even though there have been six studies employing SDMs to predict the potential distribution of *P. parva* , no study has yet used ensemble models to forecast its global distribution under future climate change (Chen 2008; Zhang et al. 2014; Fletcher et al. 2016; Artaev 2023; Tabasinezhad et al. 2023; Fang 2024), which obviously handicaps the control of this fish's further spread. Thus, this study utilizes ensemble models to predict the global distribution of *P. parva* under future climate change scenarios.

In this study, we aimed to: (1) identify the key predictive variables influencing the distribution pattern of *P. parva* ; (2) investigate the global distribution pattern of *P. parva* under current climate conditions; (3) simulate changes in the potential distribution of *P. parva* during two future periods; and (4) provide reliable recommendations for the management of *P. parva* .

## 2 Materials and Methods

### 2.1 Species Occurrence Data

Global occurrence data of *P. parva* were collected from Global Biodiversity Information Facility (GBIF; https://www.gbif.org/), FishBase (https://fishbase.de/), and literature databases (*n* = 921), containing China National Knowledge Infrastructure (CNKI; https://www.cnki.net/), Web of Science (WOS; https://www.webofscience.com/), and Elsevier (https://www.sciencedirect.com/). All data were collected up to December 2024.

**FIGURE 1:**
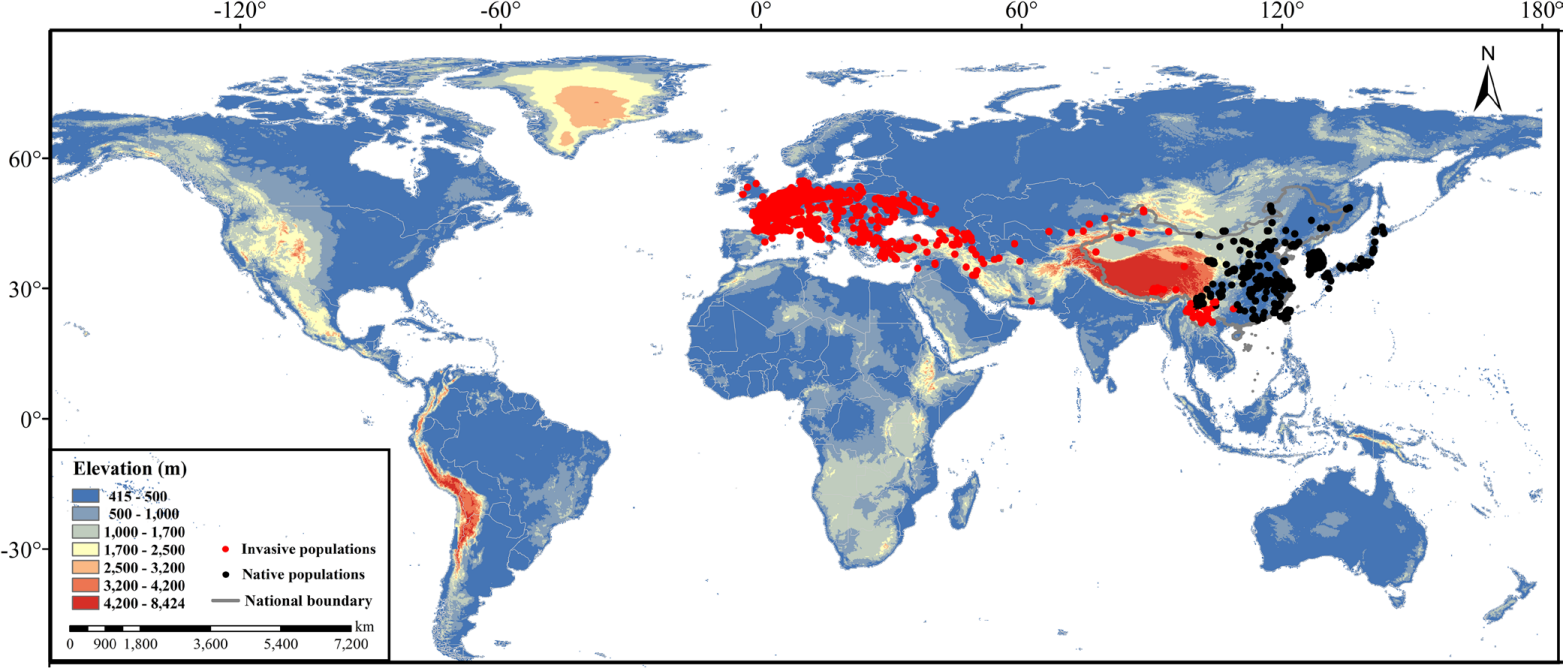
The distribution of native (black dots) and invasive (red dots) populations of *P. parva* .

After gathering all the data, obviously incorrect data records were excluded. Then, a spatial thinning approach was used to make sure that each environmental grid cell (spatial resolution of 2.5 arc minutes) contains at most one presence record (Zhao 2024). Given the global‐scale prediction objectives, harmonizing data resolution to 2.5 arc minutes was fundamental to ensuring data consistency and predictive accuracy. First, unifying all input data to a 2.5‐arc minute grid minimized spatial heterogeneity errors, avoiding distortions from disparate resolutions. Second, it provided sufficient granularity to capture large‐scale ecological patterns essential for global habitat predictions while significantly reducing computational complexity. Correspondingly, 4495 records were retained (Figure 1).

Previous studies have demonstrated that presence‐absence datasets yield higher predictive accuracy than presence‐only datasets in species distribution modeling (Elith et al. 2006). Given the practical challenges in obtaining verified absence data at global scales, 4495 pseudo‐absence points were randomly generated, excluding grid cells with known presence records. The combined presence‐pseudoabsence dataset was subsequently served as the input for ensemble modeling.

### 2.2 Bioclimatic Variables

**FIGURE 2:**
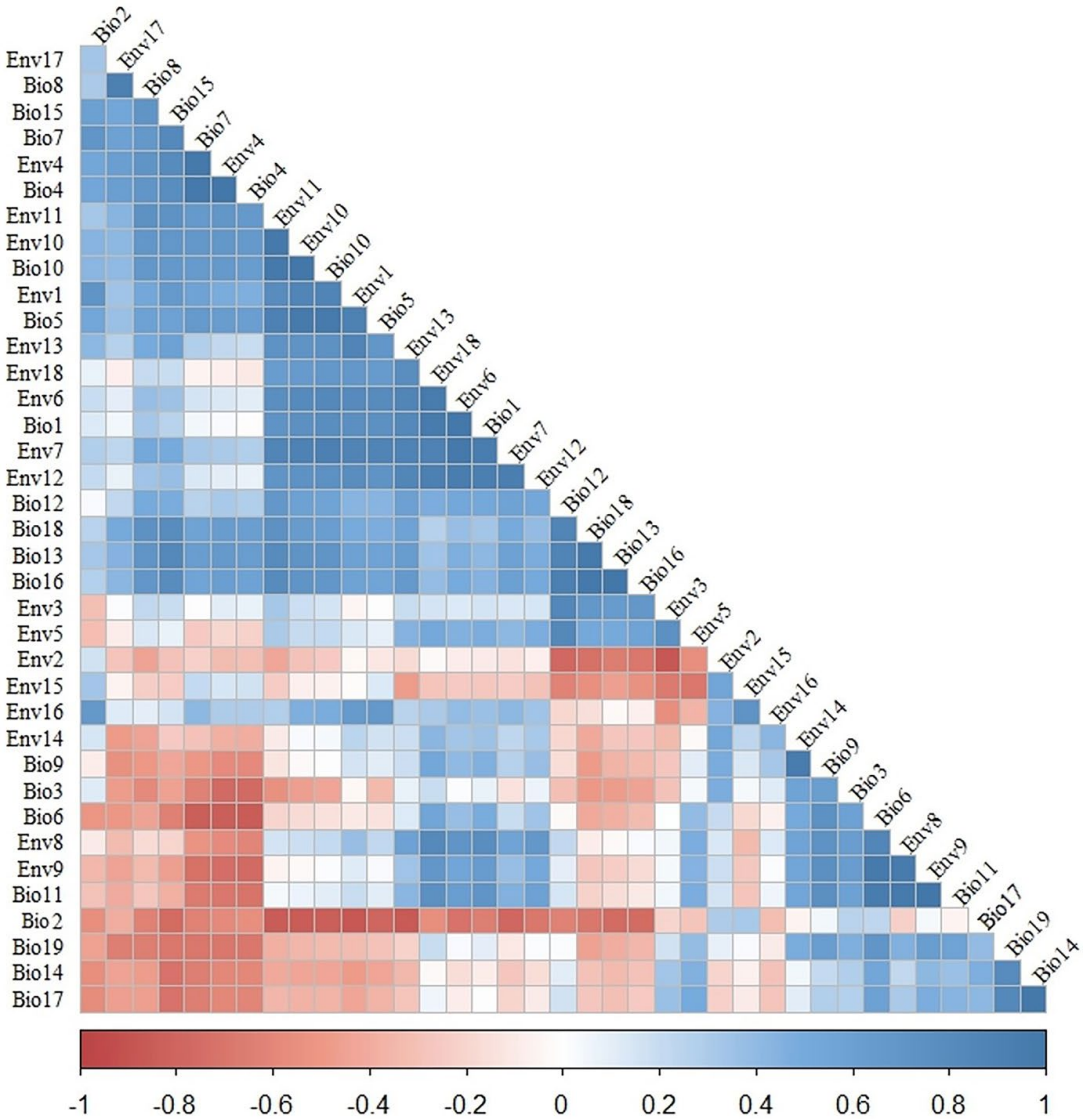
Correlation analysis between bioclimatic variables.

According to relevant experts and preliminary experiment, *P. parva* is primarily driven by temperature and habitat connectivity, and when conducting SDM predictions, including too many predictor variables will significantly decrease the model's generalization ability (Gozlan et al. 2010; Warren and Seifert 2011). So we chose two sets of bioclimatic variables (Table 1) (Dong et al. 2020; Zhao 2024). (1) WorldClim dataset (https://worldclim.org/), which was widely used in ecological modeling processes; (2) ENVIREM dataset (http://envirem.github.io/), which can enhance model prediction accuracy (Zhao 2024). All variables were resampled to 2.5 arc‐minute resolution to ensure the accuracy and performance of our model. As high correlation among environmental variables can lead to model overfitting and reduced prediction accuracy, to mitigate collinearity and improve model computational efficiency, variables were screened based on the species' biological characteristics and Pearson correlation coefficients (Guisan and Thuiller 2005; Fletcher et al. 2016). When |*r*| ≥ 0.8, the variable considered more biologically relevant to the species' life history was retained (Figure 2) (Wang et al. 2019; Aidoo et al. 2023).

**TABLE 1:** The 37 bioclimatic variables used in the present study, and those with bold fonts were for subsequent modeling.

Code	Description	Unit
Bio1	Annual Mean Temperature	°C
Bio2	Mean monthly temperature difference	°C
**Bio3**	Isothermality (Bio2/Bio7) (*100)	**/**
Bio4	Temperature Seasonality (standard deviation*100)	/
Bio5	Max Temperature of Warmest Month	°C
Bio6	Min Temperature of Coldest Month	°C
Bio7	Temperature Annual Range (Bio5‐Bio6)	°C
**Bio8**	**Mean Temperature of Wettest Quarter**	**°C**
Bio9	Mean Temperature of Driest Quarter	°C
Bio10	Mean Temperature of Warmest Quarter	°C
Bio11	Mean Temperature of Coldest Quarter	°C
Bio12	Annual Precipitation	mm
Bio13	Precipitation of Wettest Month	mm
**Bio14**	**Precipitation of Driest Month**	**mm**
Bio15	Variation coefficient of precipitation	/
Bio16	Precipitation of Wettest Quarter	mm
Bio17	Precipitation of Driest Quarter	mm
**Bio18**	**Precipitation of Warmest Quarter**	**mm**
Bio19	Precipitation of Coldest Quarter	mm
Env1	Annual Potential Evapotranspiration	mm/year
**Env2**	**Thornthwaite Aridity Index**	**/**
Env3	Climatic Moisture Index	/
Env4	Continentality (meanTempWarmest—meanTempColdest)	°C
Env5	Emberger's Pluviothermic Quotient	/
Env6	Growing Degree‐Days (GDD > 0°C)	/
Env7	Growing Degree‐Days (GDD > 5°C)	/
**Env8**	**Max Temperature of Coldest Month**	**°C**
Env9	Mean Temperature of Coldest Month	°C
Env10	Mean Temperature of Warmest Month	°C
**Env11**	**Min Temperature of Warmest Month**	**°C**
Env12	The Number of Months with Mean Temperature Greater than 10°C	mouths
Env13	Mean Monthly PET of Coldest Quarter	mm/month
**Env14**	**Mean Monthly PET of Driest Quarter**	**mm/month**
**Env15**	**Monthly Variability in PET**	**mm/month**
Env16	Mean Monthly PET of Warmest Quarter	mm/month
Env17	Mean Monthly PET of Wettest Quarter	mm/month
Env18	Compensated Thermicity Index	/

*Note:* / means dimensionless.

Future bioclimatic data for the 2050s (2041–2060) and 2070s (2061–2080) were obtained from WorldClim. To represent future climate scenarios, we utilized Global Climate Models (GCMs) released under the Coupled Model Intercomparison Project Phase 6 (CMIP6) framework. To address the inconsistencies observed among different GCMs in predicting the potentially suitable habitats of *P. parva* under future climate scenarios, and to enhance prediction accuracy, this study employed three high‐performance GCMs (FIO‐ESM‐2‐0, MPI‐ESM1‐2‐HR, and BCC‐CSM2‐MR) (Xian et al. 2023; Zhang et al. 2024). These models were driven by the Shared Socioeconomic Pathways (SSPs) developed by the IPCC (Intergovernmental Panel on Climate Change). The two most extreme pathways, SSP1‐2.6 (low emissions) and SSP5‐8.5 (high emissions), were selected to simulate the different CO_2_ emission levels in the future.

### 2.3 Ensemble Species Distribution Modeling

To enhance stability and accuracy of predictions, ensemble modeling was implemented (Dong et al. 2020; Zhao 2024). We integrated six prominent algorithms (ANN, FDA, GAM, GBM, MARS, and RF) from contemporary ecological niche modeling (Table 2). The presence‐pseudoabsence dataset was partitioned into training (80%) and validation (20%) subsets, with the former used for parameter calibration and feature extraction, while the latter was used independently for assessing generalization capacity (Avise et al. 1998). To avoid potential errors caused by dataset partitioning and enhance the reliability of our results, each algorithm underwent 10 replicate runs to generate 10 distinct base models (Dong et al. 2020).

**TABLE 2:** The six algorithms and their abbreviations for building ensemble model.

Number	Model	Code
1	Artificial Neural Network	ANN
2	Flexible Discriminant Analysis	FDA
3	Multiple Adaptive Regression Splines	MARS
4	Random Forest	RF
5	Generalized Additive Model	GAM
6	Generalized Boosting Model	GBM

The Area Under Curve (AUC) is selected as our evaluation metric. AUC is a threshold‐independent value quantifying model performance by calculating the area under the receiver operating characteristic (ROC) curve (Fielding and Bell 1997; Xiang et al. 2023). Due to its threshold independence, AUC is more objective and reliable than other performance metrics like true skill statistic (TSS). Even more, there is evidence showing that TSS is in significant correlation with AUC to some extent (Allouche et al. 2006). AUC ranges from 0 to 1, with values closer to 1 indicating better performance (Table 3) (Wu 2023). Based on the facts mentioned above, AUC is a key metric in ensemble modeling, typically used to pick out high‐performing sub‐models (AUC > 0.9) for weighted averaging and to evaluate model generalization (Duque‐Lazo et al. 2018; Wu 2023; Zhao 2024).

**TABLE 3:** Model performance evaluation criteria.

Evaluation criteria	AUC
Poor	0.5–0.7
Ordinary	0.7–0.8
Good	0.8–0.9
Excellent	0.9–1.0

The output of the ensemble model is a continuous raster with values from 0 to 1 (higher values indicating better suitability). According to the magnitude of these values, we classified the suitable habitat areas as fellows: unsuitability (0–0.2), low suitability (0.2–0.4), medium suitability (0.4–0.6), and high suitability (0.6–1.0) (Zhang et al. 2018; Li et al. 2024). The centroid of the entire suitable area (0.2–1.0) was calculated to analyze directional shifts (latitude/longitude) under different future periods, GCMs, and SSPs. This study generates predictive maps for each future climate scenario, preserving critical climate variability information and preventing underestimation of potential risks in different scenarios, thereby providing scientific support for decision‐making. All data analysis and visualization in this study were performed using R 4.4.1 (R Core Team 2022) and ArcGIS 10.8 (ESRI 2018).

## 3 Results

### 3.1 Model Performance

**FIGURE 3:**
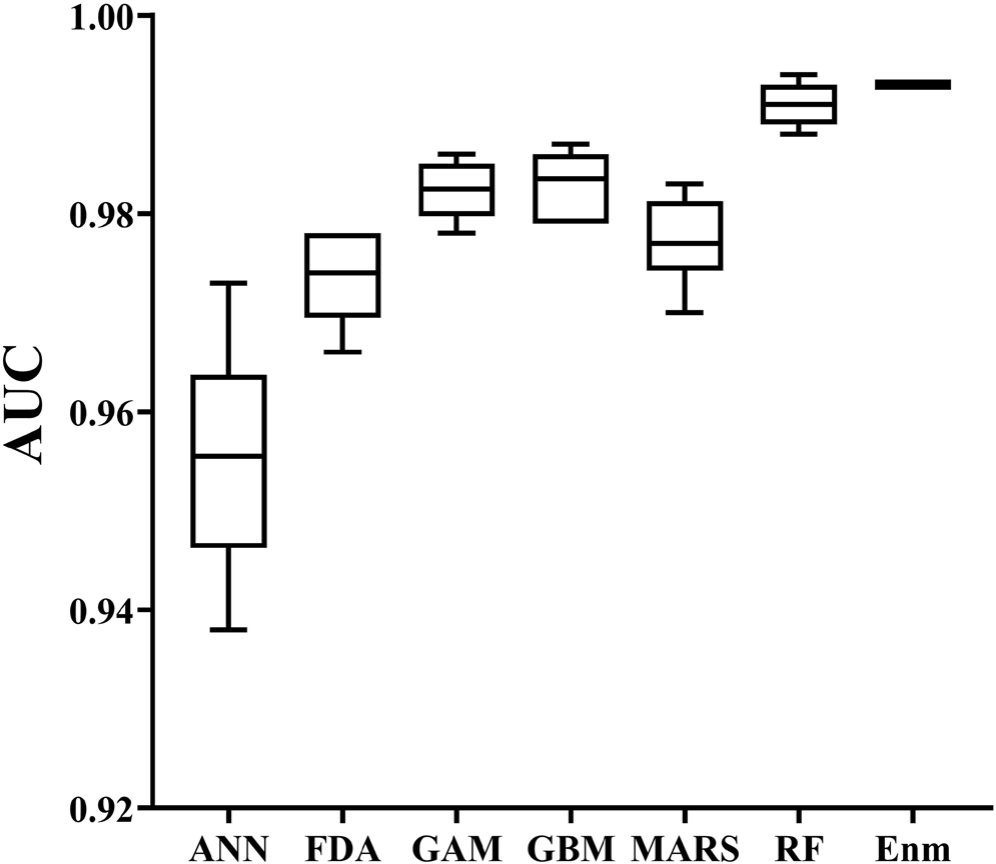
AUC values for different algorithms; Enm is the abbreviation of ensemble model. ANN, Artificial Neural Network; FDA, Flexible Discriminant Analysis; GAM, Generalized Additive Model; GBM, Generalized Boosting Model; MARS, Multiple Adaptive Regression Splines; RF, Random Forest; Enm, Ensemble model.

The ensemble model outperforms all individual algorithms with the highest AUC score of 0.993, and was classified as “Excellent” based on the criteria in Table 3. Although there were some differences between the 10 underlying models in the same algorithm, the predictive ability of the six modeling algorithms was consistently excellent (Figure 3). Based on the median AUC score, RF was the most predictive algorithm (AUC = 0.991), followed by GBM (0.983), GAM (0.982), MARS (0.977), FDA (0.973.), and ANN (0.956) (Table 4).

**TABLE 4:** Prediction performance of the six different algorithms.

Model	AUC
ANN	0.956 ± 0.011
FDA	0.973 ± 0.005
GAM	0.982 ± 0.003
GBM	0.983 ± 0.003
MARS	0.977 ± 0.004
RF	0.991 ± 0.002
Enm	0.993

*Note:* Values were shown as mean ± standard deviation; Enm is the abbreviation of ensemble model.

### 3.2 Variable Importance and Response Curves

**FIGURE 4:**
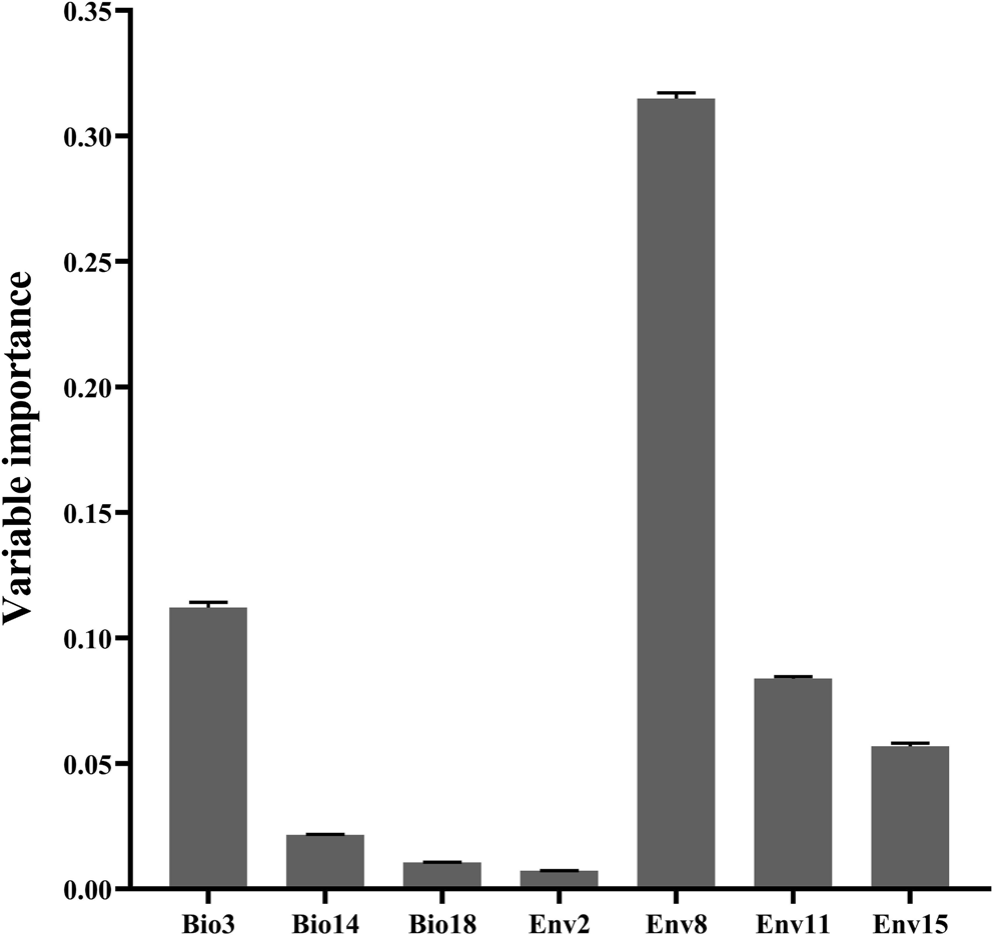
Importance of various bioclimatic variables.

We screened 37 bioclimatic variables by combining the life‐history traits of *P. parva* and Pearson correlation coefficients between variables. Finally, 9 bioclimatic variables were retained for subsequent modeling, which were Isothermality (Bio3), Mean Temperature of Wettest Quarter (Bio8), Precipitation of Driest Month (Bio14), Precipitation of Warmest Quarter (Bio18), Thornthwaite Aridity Index (Env2), Max Temperature of Coldest Month (Env8), Min Temperature of Warmest Month (Env11), Mean Monthly PET of Driest Quarter (Env14) and Monthly Variability in Potential Evapotranspiration (PET) (Env15). Ensemble model indicated that Env8 and Bio3 were the two most influential factors for predicting the potential habitat suitability of *P. parva* , with respective importance value of 0.315 ± 0.004 and 0.112 ± 0.005 (Figure 4 and Table 5).

**TABLE 5:** Importance and contribution of various bioclimatic variables.

Variables	Importance	Contribution rate	Cumulative contribution rate
Env8	0.3149	51.36%	51.36%
Bio3	0.1121	18.28%	69.64%
Env11	0.0838	13.67%	83.31%
Env15	0.0569	9.28%	92.59%
Bio14	0.0216	3.52%	96.11%
Bio18	0.0105	1.72%	97.83%
Env2	0.0071	1.16%	98.99%
Bio8	0.0033	0.55%	99.54%
Env14	0.0028	0.46%	100.00%

**FIGURE 5:**
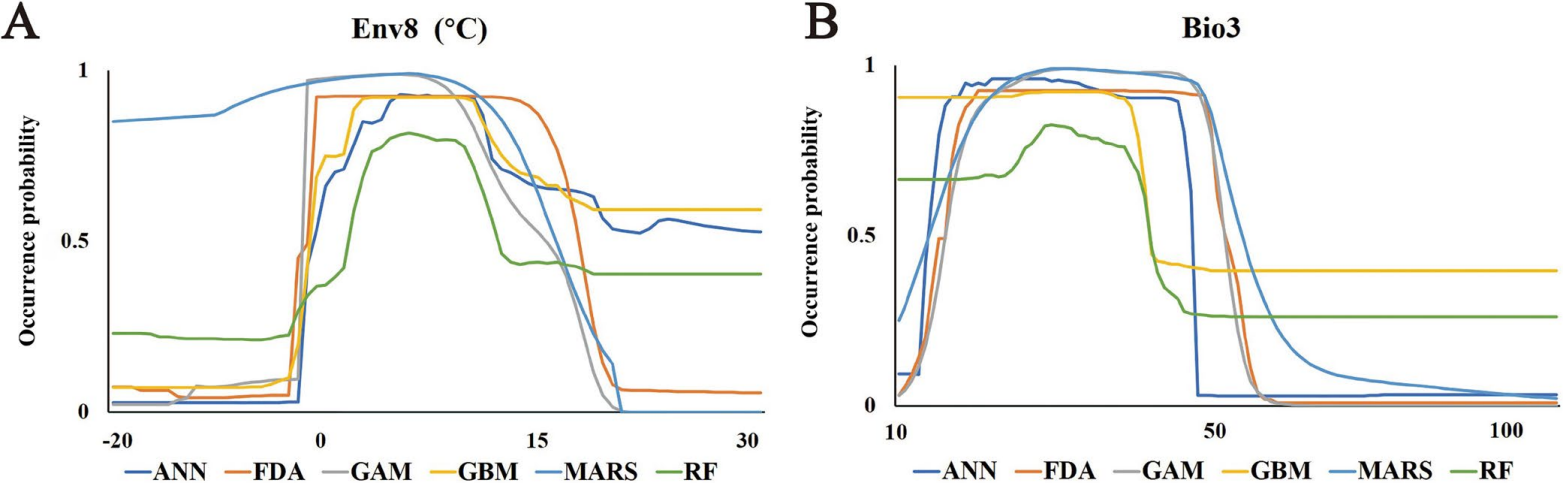
Response curves for the two most important bioclimatic variables. (A): Max Temperature of Coldest month (Env8); (B): Isothermality (Bio3).

Response curves for the two most important variables are shown in Figure 5. Roughly, the two are similar in outline. Specifically, for Env8, the occurrence probability of *P. parva* increased with temperature from −3°C to 0°C and began to decrease from about 12°C and remained stable above 24°C (Figure 5A); for Bio3, the occurrence probability of *P. parva* increased within the range of 13–23, then witnessed a plummet at about 50, and finally stabilized above 60 (Figure 5B).

### 3.3 Potential Suitable Habitat

**FIGURE 6:**
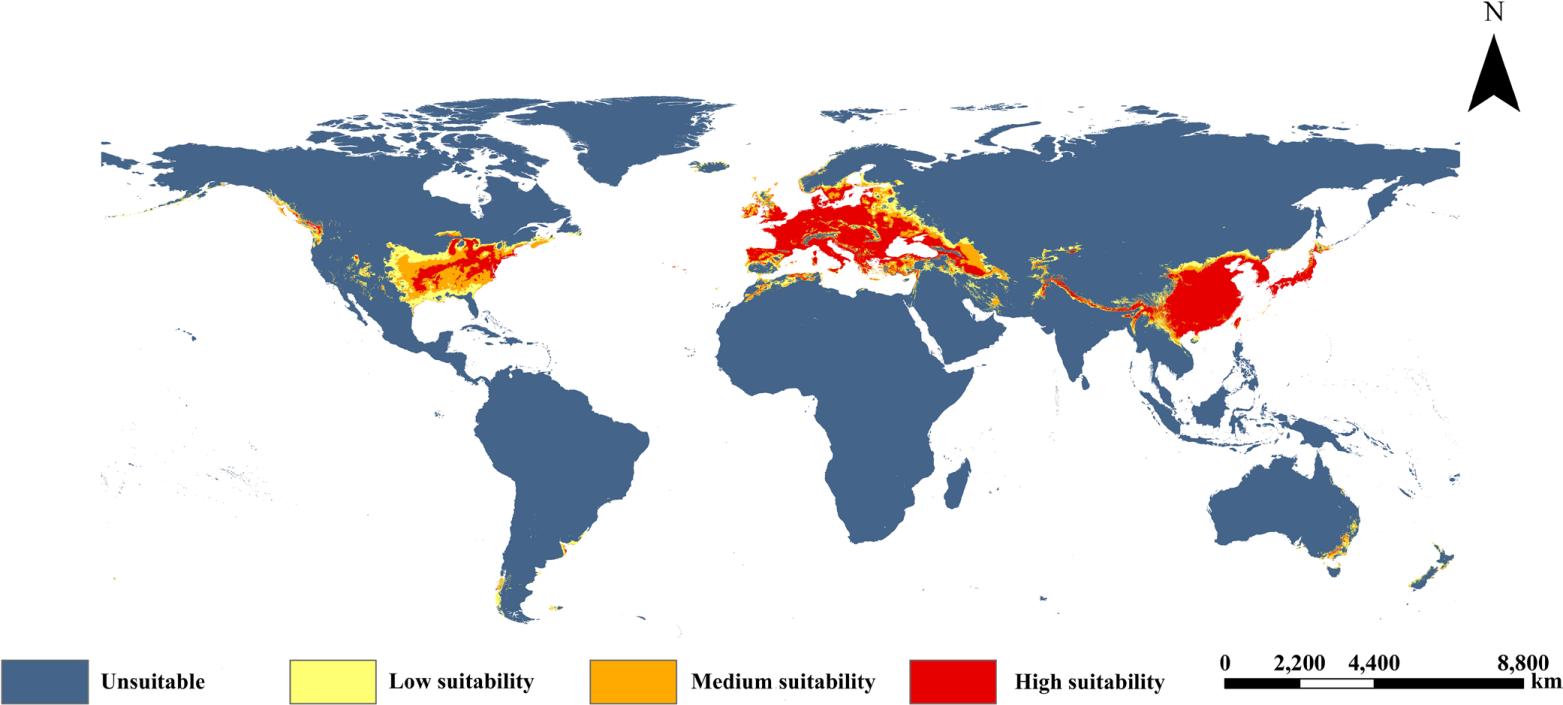
The distribution of potential suitable habitat for *P. parva* in the current period.

**FIGURE 7:**
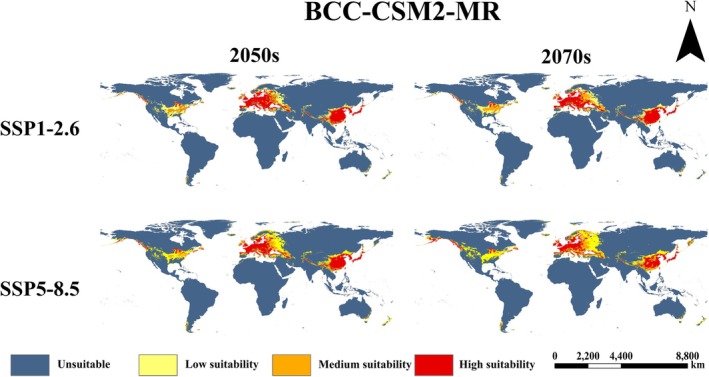
The distribution of potential suitable habitat for *P. parva* in the BCC‐CSM2‐MR model.

**FIGURE 8:**
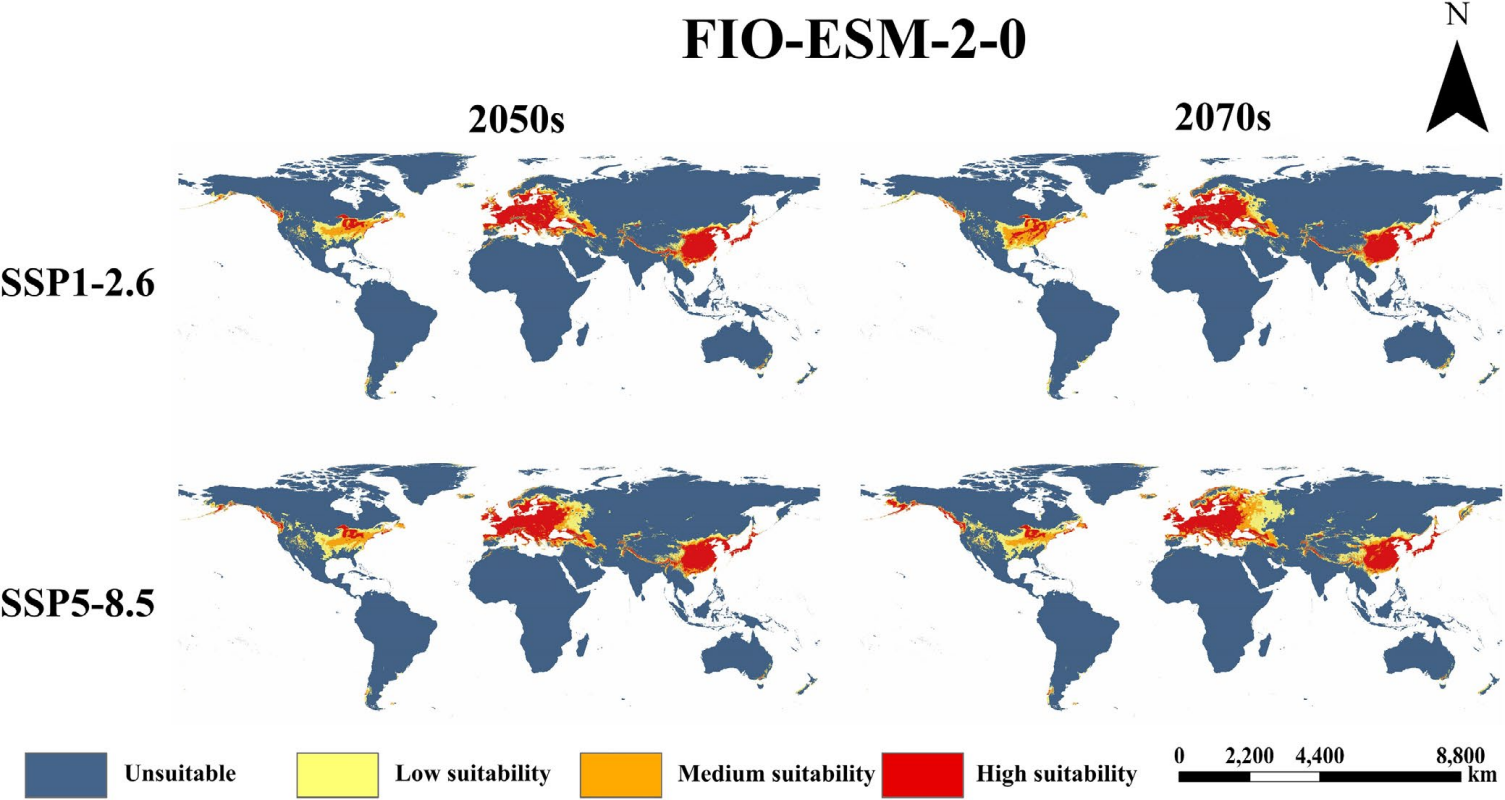
The distribution of potential suitable habitat for *P. parva* in the FIO‐ESM‐2‐0 model.

**FIGURE 9:**
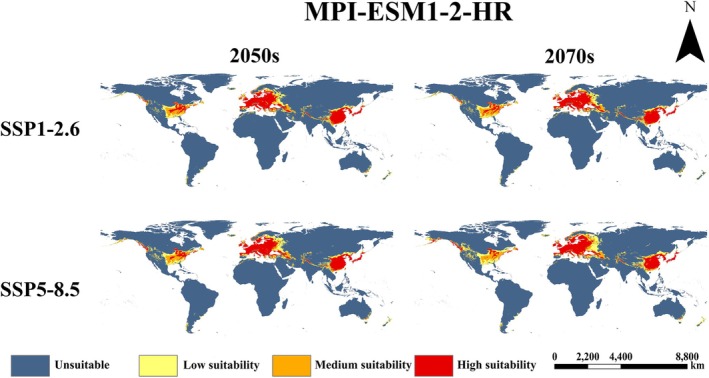
The distribution of potential suitable habitat for *P. parva* in the MPI‐ESM1‐2‐HR model.

The ensemble model successfully predicted the current global potential suitable habitat distribution of *P. parva* (Figure 6). The prediction shows that the suitable areas are concentrated in Eurasia and North America. Within the native range, eastern China, Japan, North Korea, and South Korea remain highly fit. Europe is the primary invaded continent; its highly appropriate areas include France, United Kingdom, northern Spain, Italy, Ireland, Germany, extending north to 62° N. As for North America, the fish's potential suitable habitat is scattered mainly in its southeast. Predictions for 12 future climate scenarios are shown in Figures 7, 8, 9.

**FIGURE 10:**
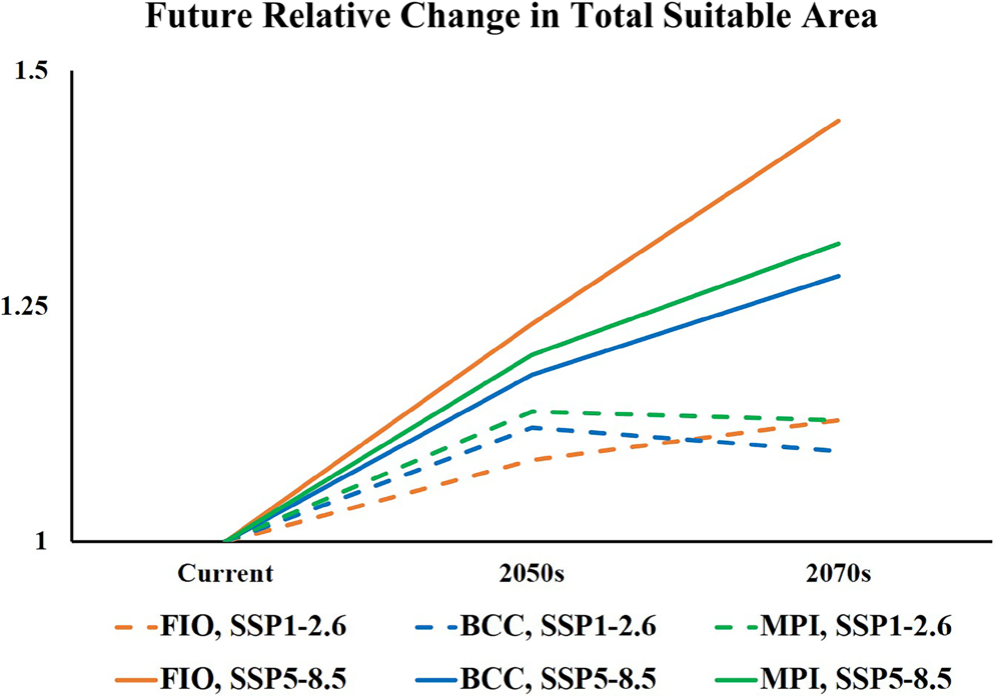
Relative changes in the potential suitable habitat of *P. parva* in future periods.

Compared to the current period, future projections indicate a northward expansion and overall increase in suitable area for *P. parva* (Figure 10). Highly suitable area remains concentrated in East Asia and Europe in the future. Medium and low suitability areas expand around these core zones, extending further north and south. Predictions varied among scenarios. Expansion magnitude was generally greater in the 2070s than in the 2050s. Among selected GCMs, FIO‐ESM‐2‐0 has the largest predicted expansion (Figure 8), followed by MPI‐ESM1‐2‐HR (Figure 9), with BCC‐CSM2‐MR showing the smallest expansion (Figure 7). Under the high‐emission SSP5‐8.5 pathway, expansion was consistently greater than the low‐emission SSP1‐2.6 pathway across all models and periods.

**FIGURE 11:**
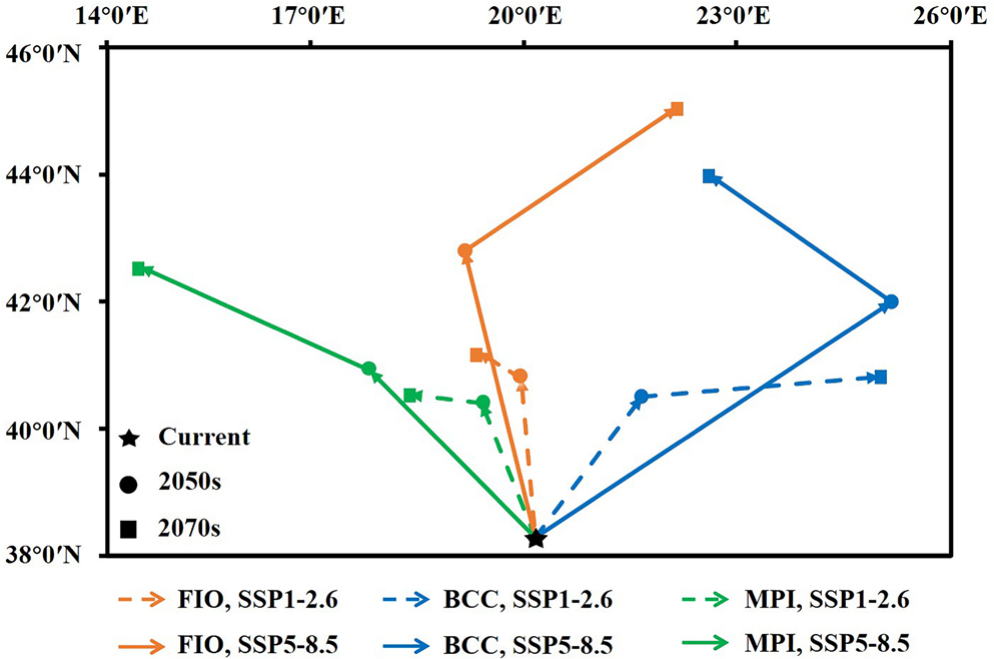
The transfer paths of the centroid of *P. parva's* potential suitable habitat under different climate scenarios.

The centroid of potential suitable area changed under different scenarios of future climate, showing an overall trend of northward shift. However, there are differences in the east–west shift predicted by different climate models. Specifically, during the SSP5‐8.5 pathway at 2070s, the MPI‐ESM1‐2‐HR model shows the largest expansion of the centroid towards west while the BCC‐CSM2‐MR model shows the largest eastward shift (Table 6 and Figure 11).

**TABLE 6:** Latitude and longitude of the center mass of *P. parva* in future climate patterns.

Periods	Longitude	Latitude
Current	20.08 E	38.29 N
FIO‐ESM‐2‐0, SSP1‐2.6, 2050s	19.89 E	40.79 N
FIO‐ESM‐2‐0, SSP1‐2.6, 2070s	19.28 E	41.21 N
FIO‐ESM‐2‐0, SSP5‐8.5, 2050s	19.08 E	42.80 N
FIO‐ESM‐2‐0, SSP5‐8.5, 2070s	22.11 E	45.05 N
BCC‐CSM2‐MR, SSP1‐2.6, 2050s	21.60 E	40.50 N
BCC‐CSM2‐MR, SSP1‐2.6, 2070s	24.99 E	40.81 N
BCC‐CSM2‐MR, SSP5‐8.5, 2050s	25.16 E	41.99 N
BCC‐CSM2‐MR, SSP5‐8.5, 2070s	22.56 E	44.00 N
MPI_ESM1_2_HR, SSP1‐2.6, 2050s	19.33 E	40.40 N
MPI_ESM1_2_HR, SSP1‐2.6, 2070s	18.30 E	40.54 N
MPI_ESM1_2_HR, SSP5‐8.5, 2050s	17.73 E	40.91 N
MPI_ESM1_2_HR, SSP5‐8.5, 2070s	14.46 E	42.55 N

## 4 Discussion

This study used an ensemble model to predict the potential suitable habitat of *P. parva* under future climate patterns at a global scale for the first time. This model combined this fish's current global occurrence data and future bioclimatic data, as well as integrated six well‐performed algorithms in ecological modeling (Zhao 2024). As anticipated, the ensemble model achieved high prediction accuracy, i.e., strong generalization ability, and thus provided more robust results, which confirmed that it was reliable and accurate to assess the current and future potential distribution of *P. parva* . Furthermore, by this model, we identified the key variables influencing the distribution pattern of *P. parva* and simulated its potential future invasion, thereby provided a scientific basis for proactive governmental prevention of its further introductions.

### 4.1 Variable Importance and Response Curves

Temperature has a significant influence on fish distribution, and regulates the entire stage of fish population growth and reproduction to a large extent (Campana et al. 2020; Zhu et al. 2024). Specifically, water temperature affects fish's metabolic rates, thereby feeding efficiency and then growth cycles. Concurrently, water temperature threshold also provides the accumulated temperature necessary for fish's gonadal maturation, ultimately defining species‐specific breeding seasons of this taxon. In this study, the most influential bioclimatic variables to the distribution of *P. parva* were Env8 and Bio3.

The Max Temperature of Coldest Month (Env8) represents the highest temperature recorded during the coldest month of an annual cycle, reflecting climate thresholds. As an aquatic organism, the survival and reproduction of *P. parva* are directly affected by water temperature: a too low Env8 may lead to frozen waters in the coldest month, making it unsuitable for *P. parva* to survive. From the response curve of Env8, it can be seen that when the temperature exceeds the critical threshold of 0°C, the occurrence probability of *P. parva* shows a substantial increase. Moreover, it is noteworthy that, as a species native to regions as far north as Heilongjiang of China and the Far East of Russia, *P. parva* possesses considerable cold tolerance. Furthermore, the bioclimatic variables used in this study represent atmospheric temperature, which means that the water temperature is higher than the response curve. Consequently, once the critical temperature threshold is surpassed, the probability of *P. parva* occurrence increases sharply with the rise of temperature.

Isothermality (Bio3) reflects the predictability and stability of a region's climate. Higher Bio3 indicates a greater ratio of diurnal temperature range to the annual temperature range, signifying relatively smaller annual temperature range and greater thermal stability. Generally, except for extremophiles (e.g., desert species), most organisms prefer climatically stable, suitable areas (Årevall et al. 2018; Coelho et al. 2023). Hence, a conclusion can be drawn by this study that *P. parva* may prefer waters with stable temperatures and less seasonal variation, which is consistent with previous study (Artaev 2023).

### 4.2 Potential Distribution Pattern

The current potential distribution of *P. parva* in Asia and Europe (Figure 6) by the ensemble model aligns with the range of actual sampling points (Figure 1), which are primarily located between 22°N and 55°N, and the prediction by a previous study (Zhang et al. 2014). Another important result is that, although no wild occurrences have been currently documented in North America, this study predicted there were sizable potential suitable habitats for *P. parva* in this continent, and they were larger than previous studies (Zhang et al. 2014; Fletcher et al. 2016). To make a long story short, there are many differences between the present study and the previous ones, and several factors may contribute to these differences: (1) This study employed six well‐performing ecological modeling algorithms to build an ensemble SDM. (2) This study incorporated a randomized method to generate pseudo‐absence data when modeling, which can improve the accuracy of the ensemble model. (3) This study compiled a more comprehensive dataset of *P. parva* occurrence records. Consequently, our predictions for the current and future potential distribution of *P. parva* are of higher credibility and precision.

As for the future potential distribution pattern of *P. parva* , variations exist among different GCMs. However, despite observed variations in predictions, our results reveal an overarching trend: under climate change, the potential distribution of *P. parva* will expand, with higher latitude or higher altitude regions becoming increasingly suitable for its survival. Additionally, according to the prediction, compared to the low‐emission scenario (SSP1‐2.6), the potential invaded range of *P. parva* is larger under the high‐emission scenario (SSP5‐8.5). This indicates that higher greenhouse gas emissions probably facilitate more serious invasion by *P. parva* in the future. Based on this, more attention currently should be paid on North America, a continent appearing very vulnerable to this fish's further invasion. And accordingly, appropriate management strategies should be formulated in advance.

### 4.3 Management Recommendations

*P. parva* is classified as an Invasive Alien Species of Union concern under EU Regulation, rendering its introduction, possession, trade, and transport illegal (Gozlan et al. 2010). In addition to the above controls, our suggestions are as follows: (1) Implement pre‐border risk assessment systems; (2) Develop rapid response strategies for population eradication during the initial invasion stage, and this one is particularly pressing for administrators of North America; (3) Establish ecological barriers in aquaculture water bodies; (4) Use innovative bio‐surveillance monitoring techniques like environmental DNA (eDNA) to detect potential invaders; (5) Conduct public education campaigns to enhance the identification ability of practitioners and the public regarding invasive species.

## 5 Conclusion

This study employed an ensemble SDM to predict the global potential suitable area of *P. parva* under current and future climate change scenarios for the first time. The results indicate that Env8 and Bio3 are the two most critical bioclimatic factors influencing the distribution of *P. parva* . In the current period, the primary invasion areas of *P. parva* are concentrated in East Asia, Europe, and the North American Great Lakes region within 22°N–55°N. In different future climate scenarios, the expansion magnitude of *P. parva* varies. The overall trend shows that in the future, the potential suitable areas of *P. parva* will further expand, with high‐latitude or high‐altitude regions becoming more conducive to its survival. The centroid overall shifts northward, with variations in east–west directions. Europe and North America continue to be highly potential suitable areas for *P. parva* , thus requiring attention and timely formulation of appropriate management measures. To enhance this study, other variables such as human factors, terrain, water depth, and water type can be comprehensively considered in future research.

## Author Contributions

**Hao Li:** data curation (equal), formal analysis (equal), validation (equal), writing – original draft (lead). **Wenqian Sun:** investigation (equal), methodology (equal), software (equal), visualization (equal), writing – original draft (lead). **Wen Xiong:** formal analysis (equal), supervision (equal), writing – review and editing (equal). **Tao Ju:** methodology (equal), supervision (equal), writing – review and editing (equal). **Wenhui Wang:** formal analysis (equal), writing – review and editing (equal). **Li Tang:** formal analysis (equal), writing – review and editing (equal). **Zhengxiang Wang:** funding acquisition (equal), project administration (equal), supervision (equal), writing – review and editing (equal). **Xin Gao:** formal analysis (equal), supervision (equal), writing – review and editing (equal). **Lei Pan:** formal analysis (equal), project administration (equal), supervision (equal), writing – review and editing (equal). **Xianghong Dong:** methodology (equal), software (equal), supervision (equal), writing – review and editing (equal). **Yu Peng:** project administration (equal), supervision (equal), writing – review and editing (equal).

## Funding

This work was supported by Natural Science Foundation of Hubei Province, 2022CFB329.

## Disclosure

The authors have nothing to report.

## Ethics Statement

This study did not sacrifice fish, so ethical approval was not required.

## Conflicts of Interest

The authors declare no conflicts of interest.

## Data Availability

Access to all datasets is as follows: The modeling data Bio1‐19 and Env1‐18 are respectively from WorldClim and ENVIREM. Codes for this study in R 4.3.3 are available in Dong et al. (2020). The data that support the findings of this study are openly available in Dryad at https://doi.org/10.5061/dryad.nvx0k6f5n.
